# Correction: Potential Theory for Directed Networks

**DOI:** 10.1371/annotation/6dff4052-f7c3-4b0a-88da-85cdd5d3addd

**Published:** 2013-08-02

**Authors:** Qian-Ming Zhang, Linyuan Lü, Wen-Qiang Wang, Yu-Xiao Zhu, Tao Zhou

The name of the fourth author was given incorrectly. The correct name is: Yu-Xiao Zhu. The correct citation is: "Zhang Q-M, Lü L, Wang W-Q, Zhu YX, Zhou T (2013) Potential Theory for Directed Networks. PLoS ONE 8(2): e55437. doi:10.1371/journal.pone.0055437." The correct abbreviation in contributions is: YXZ. 

